# Longitudinal Associations between School Quality in Adolescence and Cognitive Health in Older Adulthood

**DOI:** 10.1093/geroni/igaf122.112

**Published:** 2025-12-31

**Authors:** Joy Bohyun Jang, Lindsay Ryan, Megan Patrick

**Affiliations:** University of Michigan, Ann Arbor, Michigan, United States; University of Michigan, Ann Arbor, Michigan, United States; University of Michigan, Ann Arbor, Michigan, United States

## Abstract

Studies show that residing in states with higher-resourced education systems in childhood is associated with better cognitive health in older adulthood (Walsemann et al., 2023; 2024). One limitation is that state-level measures may not accurately reflect the quality of the schools that respondents attended earlier in life. Using data from a national sample and school administrative records, we examined the association between school quality in adolescence and cognitive health in older adulthood. Longitudinal data from individuals who were high school seniors in 1978-1981 and who participated in the age 60 survey in 2020-2023 were used from the Monitoring the Future (MTF) Panel study (n = 2,090). MTF first surveys high school seniors at their schools and follows up a selective sample over the course of their lives. Cognitive health was measured by self-reported poor memory at age 60 (coded as 1=poor/fair; 0=good/very good/excellent). School quality measures (student/teacher ratio at the schools, school-district level expenditure and revenue per student) were obtained from the National Center for Education Statistics (NCES). Survey-weighted logistic regression models were used. About 10% of the analytic sample reported poor memory at age 60. Regression findings show that a higher student/teacher ratio is associated with poor memory at age 60, particularly among those who attended schools in the South and among racial minority groups (i.e., other than Whites). Our findings suggest that school quality in adolescence may play an important role in cognitive health in older adulthood. We will further explore the mechanisms of school quality (e.g., neighborhood socioeconomic status).

